# Successful Treatment of Uterine Arteriovenous Malformation due to Uterine Trauma

**DOI:** 10.1155/2016/1890650

**Published:** 2016-09-06

**Authors:** Burak Karadag, Onur Erol, Ozgur Ozdemir, Aysel Uysal, Ahmet Sukru Alparslan, Cemil Gurses, Mert Koroglu

**Affiliations:** ^1^Department of Obstetrics and Gynecology, Antalya Training and Research Hospital, Antalya, Turkey; ^2^Department of Radiology, Antalya Training and Research Hospital, Antalya, Turkey

## Abstract

Uterine arteriovenous malformation (AVM) is defined as abnormal and nonfunctional connections between the uterine arteries and veins. Although the patients typically present with vaginal bleeding, some patients may experience life-threatening massive bleeding in some circumstances. The treatment of choice depends on the symptoms, age, desire for future fertility, and localization and size of the lesion; however, embolization of the uterine artery is the first choice in symptomatic AVM in patients at reproductive age with expectations of future fertility. We report a case of acquired AVM (after D/C) with an extensive lesion, which was successfully treated with bilateral uterine artery embolization (UAE).

## 1. Introduction

Uterine arteriovenous malformation (AVM) is defined as abnormal and nonfunctional connections between the uterine arteries and veins. These can be either congenital or acquired lesions (traumatic) lesions. Congenital AVMs are extremely rare conditions, whereas the incidence rate of acquired AVMs is currently increasing [1–3]. Acquired AVMs are often associated with previous uterine surgery (dilation and curettage (D/C)), therapeutic abortion, cervix or endometrial cancer, trophoblastic diseases, and direct uterine trauma and occur more frequently in women at reproductive age [4]. Typical symptom is vaginal bleeding; however, some patients may present with life-threatening massive bleeding.

We report a case of acquired AVM (after D/C) with an extensive lesion, which was successfully treated with UAE.

## 2. Case

**Figure 1:**
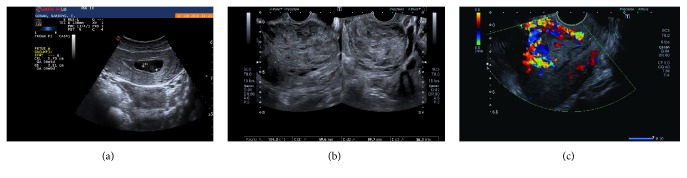
(a) Ultrasound image just before D/C shows 9-week fetus with no cardiac activity and there is no sign of uterine AVM. (b) Sagittal endovaginal image of the uterus shows 60 × 60 × 56 mm (103 cm^3^) hyperechogenic and heterogeneous mass lesion located in the anterior wall of the uterus and extending laterally at the left. (c) Color Doppler image shows multiple tortuous vessels.

**Figure 2:**
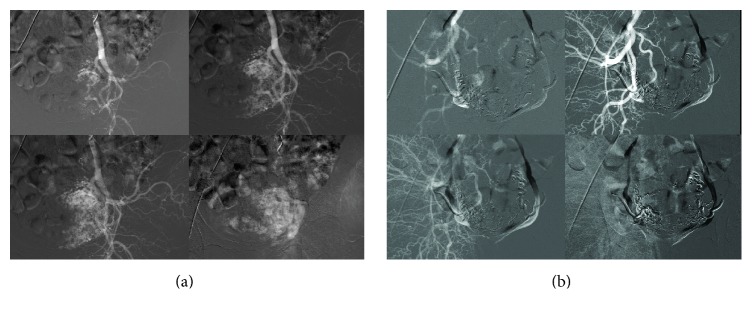
(a) Right/left internal iliac uterine artery angiography showing opacification of a slightly enlarged right uterine artery and hypervascular mass in the uterus. (b) Postembolization angiographic image.

**Figure 3:**
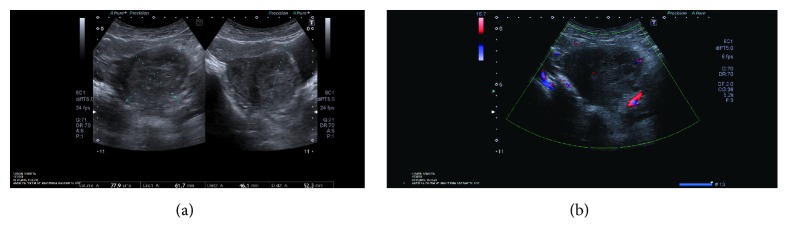
A repeat transvaginal ultrasound and color Doppler were done two months later; (a) the lesion was measuring 61 *∗* 46 *∗* 52 mm (77 cm^3^) and (b) revealed no blood flow.

A 35-year-old patient, gravida 2, para 1, abortion 1, underwent D/C at nine weeks due to missed abortion about two weeks before in another center (Figure 1). The patient underwent repeat D/C procedure at control visit one week after initial intervention in another center with a suspected hematoma; however, the procedure had been discontinued due to hemorrhage and the patient was referred to our hospital. Upon admission, Hb was 11.2 g/dL, Htc was 35.1%, and hCG was 3518 mIU/mL. There was no evidence of active vaginal bleeding. Transvaginal ultrasonography (TVUSG) revealed a 60 × 60 × 56 mm (103 cm^3^) hyperechogenic and heterogeneous mass lesion located in the anterior wall of the uterus and extending laterally at the left. There was minimal fluid collection in the endometrial cavity. The adnexa bilaterally appeared normal. Doppler ultrasonography revealed prominent venous vascular signals (Figure 1). The patient was hospitalized with the diagnosis of arteriovenous malformation. A consultation with an interventional radiologist was performed and the patient was scheduled for UAE. Bilateral UAE was performed using with the mixture of Histoacryl and Lipiodol. Pre- and postembolization images of the patient are shown in Figure 2. No complications occurred after the procedure. The patient was discharged two days after the procedure; her hCG level decreased to 1766 mIU/mL. Control Doppler USG performed one month later and revealed no blood flow and the lesion was measuring 61 *∗* 46 *∗* 52 mm (77 cm^3^) and showing shrinkage (Figure 3). hCG level was <0,5 mIU/mL.

## 3. Discussion

Uterine AVMs have an important place in gynecology practice due to risk of massive bleeding that could be life threatening in some patients. These can be either congenital or acquired (traumatic) lesions. Acquired malformations may be due to previous uterine trauma (prior pelvic operation and curettage), pathologic pregnancy-related conditions, infections, and the treatment of gestational trophoblastic disease. Congenital AVMs are considered to arise from arrested vascular embryologic development resulting in anomalous differentiation in the capillaries and abnormal communication, between arteries and veins [5]. Moreover, congenital AVMs can have multiple vascular connections and may invade surrounding structures. It is important to diagnose uterine AVM correctly and to start appropriate treatment promptly, because uterine AVM often causes life-threatening massive and persistent vaginal bleeding. The present case developed uterine AVM in a short time (about three weeks) secondary to past uterine trauma (D/C).

In today's practice, AVM is easily diagnosed using color Doppler ultrasonography [6]. But, in differential diagnosis, retained products of conception (RPOC) and gestational trophoblastic diseases (GTD) should be kept in mind because these cases may also give a hypervascular appearance with turbulent flow. BHCG levels may be helpful in diagnosis. This patient did not have an increasing pattern of hCG levels which would be expected in GTD. However, placental site trophoblastic tumor (PSTT) does not produce high levels of hCG and instead produces human placental lactogen (hPL) [7].

However, the hPL levels were not measured in this patient because GTD and PSTT were not considered in the differential diagnosis. The hCG values in the presented case were decreasing and this was thought to be due to abortion. If the diagnosis is still not certain; MR angiography is a useful diagnostic tool in elucidating the relation of AVMs to the neighboring organs and differentiating these lesions from gestational trophoblastic diseases [1].

The treatment changes depending on the age, desire for future fertility, localization, and size of the lesion. The mainstay for management of uterine AVM has been hysterectomy or the embolization of uterine arteries. However, the uterine artery embolization (UAE) remains the first choice of treatment in women at reproductive age having expectation of future fertility [8]. Whether this procedure is safe for women desiring future fertility is controversial; however, women who become pregnant after UAE are at risk of malpresentation, caesarean delivery, preterm birth, and postpartum hemorrhage [9].

Some studies have suggested that conservative approach would be appropriate in asymptomatic patients [10, 11]. Some reports have mentioned the use of methylergonovine maleate, gonadotropin releasing hormone analogues, and danazol in the treatment of patients with mild hemorrhage [12–14]. There is currently no clear agreement on the treatment of asymptomatic uterine AVMs.

In conclusion, AVMs are rare and dangerous clinical entities; their management is complicated and necessitates a high level of suspicion. Usually, these lesions are present in young women, with a previous history of spontaneous abortions. The patient presented in this case report had AVM that developed secondary to previous uterine surgery and underwent repeat intervention during control visit with suspicion of hematoma and retained products of conception; however, this final intervention resulted in an abundant bleeding. It must be kept in mind that patients may develop AVM following evacuation of uterus for pregnancy loss and the possibility of AVM must be considered in differential diagnosis.
